# Psychometric properties of the Persian version of social anxiety questionnaire for adults (SAQ-A30)

**DOI:** 10.1186/s12955-020-01457-2

**Published:** 2020-06-29

**Authors:** Mahdieh Mosarezaee, Azadeh Tavoli, Ali Montazeri

**Affiliations:** 1 Faculty of Psychology, Islamic Azad University, Science and Research Branch, Tehran, Iran; 2 Department of Psychology, Faculty of Educational Science and Psychology, Alzahra University, Tehran, Iran; 3 Population Health Research Group, Health Metrics Research Center, Iranian Institute for Health Sciences Research, ACECR, Tehran, Iran; 4 Faculty of Humanity Sciences, University of Science &Culture, ACECR, Tehran, Iran

**Keywords:** Social anxiety, Social phobia, Anxiety, Psychometric, Iran

## Abstract

**Background:**

The Social Anxiety Questionnaire for Adults (SAQ-A30) is a newly developed instrument for measuring social anxiety. This study aimed to examine the psychometric properties of the SAQ-A30 in Iran.

**Methods:**

The English version of the SAQ-A30 was translated into Persian using forward-backward procedure. The questionnaire was administered to a sample of university students. In addition they completed two other standard questionnaires namely the Brief Fear of Negative Evaluation-Straightforward version **(**BFNE-S) and the Social Phobia Inventory (SPIN). Validity was assessed using discriminant analysis, and explanatory factor analysis. In addition the correlation between SAQ-A30, the BFNE-S, and the SPIN was assessed to examine convergent validity. Finally the Cronbach’s alpha coefficient was used to examine internal consistency.

**Results:**

In all 299 students took part in the study. The mean age of participants was 23.6 (SD = 4.2) years. The analysis showed that the SAQ-A30 discriminated well between men and women where women significantly scored higher than male respondents as expected (*P* = 0.003). The exploratory factor analysis revealed a five-factor structure for the questionnaire that jointly explained 53.3% of variance observed. The results from convergent validity showed significant correlation between the SAQ-A30, the SPIN (*r* = 0.66, *P* < 0.001), and the BFNE-S (*r* = 0.5, *P* < 0.01). The internal consistency (to assess reliability) was satisfactory (Cronbach^’^s alpha coefficient = 0.92).

**Conclusion:**

The preliminary findings from this study indicated that the Persian version of SAQ-A30 is a valid instrument for measuring social anxiety in Iran. However, further psychometric evaluation of the questionnaire is recommended.

## Background

Social anxiety disorder (SAD) is a persistent and intense fear that one might experience when exposed to unfamiliar people, or performing social activities or social performances [1]. The SAD is a highly prevalent anxiety disorder with lifetime prevalence between 3 and 20% in different countries [1–6]. Findings from studies, although not consistent, indicate that gender difference exist on suffering from social anxiety. Studies have reported that women suffer significantly higher than men [1, 7–9] while others reported conversely [10, 11].

There are currently many popular questionnaires for measuring social anxiety disorder including the Liebowitz Social Anxiety Scale-LSAS [12], the Social Phobia and Anxiety Inventory-SPAI [13], the Social Phobia Scale-SPS [14], the Social Interaction Anxiety Scale-SIAS [14], and the Social Phobia Inventory-SPIN [15]. Although these questionnaires were frequently used for evaluating social anxiety in clinical and research settings, the existing instruments have a number of limitations. For instance most of these questionnaires are not empirically developed and rather they derived and conformed to a selection of items from existing instruments. The SPIN was developed based on a prior inventory namely the Brief Social Phobia Scale-BSPS [15, 16] or initial item pool of the SPAI was made up from available inventories, diagnosis criteria in the DSM-III and -IV and a patient population [13]. In addition many of the existing instruments vary in their factor structure. For example, studies reported very different factor structure for the LSAS. The authors reported three factors for the instrument [17], while others reported four [18], five [19], or even eight factors [20]. Similar inconsistent findings in factor solutions have been reported for other social anxiety/phobia measures, such as the SPIN [15, 21, 22], and the SPAI [13, 23]. Additionally, most self-reported instruments on social anxiety have been developed within English-speaking cultures, primarily North America, the United Kingdom and Australia. Finally, the samples used in these studies have often been small, e. g; The LSAS developed as clinician-administered measure based on a small sample size [12].

The SAQ-A as the first extensive version of the new self-report instrument, designed to assess social anxiety. It composed from 516 items derived from an initial pool of more than 10,000 social situations that elicited anxiety, stress, sadness or anger. It was applied to a large number of people from 10 Latin American countries and Spain and based on the objective statistical reduction of the initial scale, its revised version with 6 factors and 72 items was introduced (SAQ-AR). The Pearson correlation of the SAQ-A with SAQ-AR was 0.98 [24–26]. The SAQ-A30 is the final version of the SAQ-A with five dimensions and 30 items, which resulted from several years of work by research team through a series of statistical and clinical analyses [27]. The SAQ-A30 has been applied only to a non-clinical sample of Spanish university students [25] and in clinical and non-clinical samples from most Latin American countries, Spain, and Portugal [27]. The SAQ-A30 originally assesses five distinct solid social dimensions that better identifies social situations that could lead to social anxiety [25]. Indeed the recognition of such situations significantly could aid to implement specific treatment for those who are suffering from social anxiety [26].

To this end considering the limitations of previous instruments, it seems that the final version of the SAQ (SAQ-A30) is a good questionnaire for measuring social anxiety. In addition it is important to note that since the diagnosis of social anxiety has been changed (according to the DSM-5) and ‘generalized’ subtype is replaced by ‘performance situations’ subtype, none of the existing instruments reflect such a change except the social anxiety questionnaire [27]. Thus this study performed to translate and validate this new instrument (SAQ-A30) for the assessment of social anxiety in Iran. Currently the prevalence of social anxiety in Iran is ranging from 10 to 40% [28–32].

## Methods

### Translation

Forward-backward translation procedure was applied to translate the questionnaire from English into Persian. First two independent translators not connected to the study did the forward translation. The research team then provided a forward consolidated Persian version of the questionnaire. Accordingly two independent translators back translated the consolidated version into English and the research team provided a backward consolidated version. The consolidated backward version then was checked against the English version [25]. Finally after a few minor corrections by the research team (including a coordinator and two psychologists), the final Persian version of the questionnaire was provided. The English version of the questionnaire was provided by Caballo et al. when reporting the validation study for the original Spanish language and indicated that the questionnaire could be used for clinical and research purposes without previous authorization [25].

### Participant and data collection

In a cross-sectional study a sample of 299 university students was entered into the study. Students were **s**elected on voluntary basis by convenience sampling from different faculties of Tarbiat Modares, Tehran University and Science and Technology universities in Tehran, Iran. After obtaining written informed consent from all the study participants, the purpose and the procedure were explained to them. All participants completed brief background information (age, gender, discipline, marital status, education and job) along with the Iranian version of SAQ-A30, the SPIN, and the BFNE-S. To conduct the study the authorities of these three public universities granted permission to collect data. 

### Measures

#### The social anxiety questionnaire for adults (SAQ-A30)

The SAQ-A30 consists of 30 items with 5 dimensions: 1) Speaking in public/ talking with people in authority, 2) Interaction with the opposite sex, 3) Assertive expression of annoyance, disgust or displeasure, 4) Criticism and embarrassment, 5) Interactions with strangers. Each dimension includes 6 items and each item is rated on a 5-point Likert scale ranging from 1 (Not at all or very slight) to 5 (Very high or extremely high) level of unease, stress or nervousness [25].

#### The social phobia inventory (SPIN)

The SPIN is a 17- items questionnaire with 3 subscales including: fear, avoidance and physiological symptoms. Scores for each item are rated from 0 (Not at all) to 4 (Extremely). The Iranian versions of the SPIN showed acceptable psychometric properties and are well documented [33, 34].

#### The brief fear of negative evaluation-straight forward items (BFNE-S)

The BFNE-S is used for measuring fears of negative evaluation and includes 8 straightforwardly worded items from the BFNE [35]. Each item is rated on a 5-point Likert scale ranging from 0 (Not at all) to 4 (Extremely). The Iranian version of the Fear of Negative Evaluation-Straightforward items (BFNE-S) was administered by a sample of 150 students in the Payam Noor University in Tehran, Iran. A total of students completed a brief demographic questionnaire (age, gender, marital status, year in college), the Social Phobia Inventory (SPIN) and the BFNE-S. The Iranian version of the BFNE-S showed high internal consistency (α = 0.89). Satisfactory convergent and discriminant validity of the questioner also was established. The BFNE-S was related to the SPIN (0.58, *p* < 0.0001). The BFNE-S score was higher in women (*p* = 0.008). The result of the principle axis factoring analysis showed a unitary factor solution accounting for 58.5% of the variance observed. The factor loadings ranged from 0.66 to 0.86 (personal communication).

### Statistical analysis

Descriptive statistics were used to present data. Evaluating of internal consistency was assessed using the Cronbach’s alpha coefficient. Validity was assessed using known groups comparison to test how well the questionnaire discriminates between subgroups of the study sample that differed in gender. It was expected that women would be more socially anxious and score higher than men [1, 7–9]. The group comparison was performed using the independent samples t-test. In addition convergent validity was carried out to demonstrate the extent to which the SAQ-A30 correlates with scores derived from the SPIN and the BFNE-S. It was expected that the SAQ-A30 would positively correlate with this measures. The Pearson correlation coefficient of 0.40 or above was considered satisfactory [36]. Furthermore the factor structure of the questionnaire was extracted by performing principal component analysis.

## Result

### The study sample

In all 299 university students were studied. The characteristics of the participants are shown in Table 1.

**Table 1:** Demographic characteristics of the study sample (*n* = 299)

	No.	%
**Age**		
Mean (SD)	23.6 (4.2)	–
Range	20–40	–
**Gender**		
Male	185	61.9
Female	114	38.1
**Marital status**		
Single	234	78.3
Married	64	21.4
Divorced	1	.3
**Education**		
Bachelor student	168	56.2
Master of arts/ SC student	104	34.8
PhD student	27	9.0
**Academic major**		
Humanity	113	37.8
Technical & Engineering	159	53.2
Medicine	2	.7
Science	22	7.4
Art	3	1.0

### Validity

Convergent validly: It was examined by performing the correlation between the Iranian versions of the SAQ-A30, the SPIN and the BFNE-S. As expected a significant positive correlation was observed. The results are presented in Table 2.

**Table 2:** The correlation between the SAQ-A30, the BFNE-S and the SPIN

	SAQ-A30	BFNE-S	SPIN
**SAQ-A30**	1		
**BFNE-S**	0.50	1	
**SPIN**	0.66	0.63	1

All *p* values less than < 0.01

#### Discriminant validity

It was assessed by comparing the SAQ-A30 scores between male and female participants. Table 3 displays the results. The scale differentiated well between two genders. As hypothesized female scored higher on the SAQ-A30 and the difference was significant.

**Table 3:** Comparison of the BFNE-S, SAQ-A30 and the SPIN among male and female students

	Male (***n*** = 185)	Female (***n*** = 114)	P*
	Mean (SD)	Mean (SD)	
Social Anxiety Questionnaire (SAQ-A30)	79.2 (19.0)	86.0 (19.1)	0.003
Brief version of the Fear of Negative Evaluation Scale- straightforward items (BFNE-S)	12.1 (7.0)	14.2 (7.1)	0.012
Social Phobia Inventory (SPIN)	14.9 (10.8)	20.0 (12.1)	< 0.0001

* Derived from t-test

#### Factor analysis

Finally principal component analysis with varimax rotation resulted in five distinct factors for the instrument including speaking in public/ talking with people in authority, interaction with the opposite sex, assertive expression of annoyance, disgust or displeasure, criticism and embarrassment, interactions with strangers. The factors jointly accounted for 53.3% of variance observed. The results are shown in Table 4.

**Table 4:** Factor analysis of the SAQ-A30

(Item’s number) Item	Factor 1	Factor2	Factor3	Factor4	Factor5
(3) Speaking in public	**0.62**	0.16	0.07	−0.06	0.02
(7) Participating in a meeting with people in authority	**0.43**	0.34	−0.04	0.43	0.11
(12) Having to speak in class, at work, or in a meeting	**0.65**	0.17	0.11	0.09	0.11
(18) Being asked a question in class by the teacher or by a superior in a meeting	**0.74**	0.09	0.04	0.21	0.14
(25) While having dinner with colleagues, classmates or workmates, being asked to speak on behalf of the entire group	**0.57**	0.24	0.19	0.21	0.05
(29) Talking to a superior or a person in authority	**0.59**	0.18	0.06	0.26	0.009
(4) Asking someone attractive of the opposite sex for a date	0.06	**0.67**	−0.05	0.38	0.19
(6) Feeling watched by people of the opposite sex	0.12	**0.64**	0.07	0.11	0.24
(20) Being asked out by a person I am attracted to	0.28	**0.72**	0.20	0.07	0.09
(23) Starting a conversation with someone of the opposite sex that I like	0.20	**0.74**	0.11	0.17	0.16
(27) Asking someone I find attractive to dance	0.25	**0.51**	0.29	0.33	−0.14
(30) Telling someone I am attracted to that I would like to get to know them better	0.20	**0.65**	0.23	0.20	−0.06
(2) Having to ask a neighbor to stop making noise	0.11	0.11	**0.37**	0.40	0.02
(5) Complaining to the waiter about my food	0.04	0.27	**0.31**	0.14	0.35
(9) Refusing when asked to do something I don’t like doing	−0.003	0.15	**0.67**	0.12	0.24
(11) Telling someone that they have hurt my feelings	0.01	0.16	**0.53**	0.32	0.30
(14) Expressing my annoyance to someone that is picking on me	0.07	0.16	**0.64**	0.10	0.40
(26) Telling someone that their behavior bothers me and asking them to stop	0.20	0.19	**0.77**	0.12	0.09
(1) Greeting someone and being ignored	0.12	0.14	0.01	**0.70**	0.05
(8) Talking to someone who isn’t paying attention to what I am saying	−0.06	0.16	0.17	**0.67**	0.11
(16) Being teased in public	0.08	0.19	0.01	**0.75**	0.06
(21) Making a mistake in front of other people	0.41	0.09	0.27	**0.51**	−0.007
(24) Being reprimanded about something I have done wrong	0.28	0.07	0.35	**0.52**	−0.08
(28) Being criticized	0.43	0.04	0.01	**0.52**	0.13
(10) Making new friends	0.25	0.04	0.24	−0.13	**0.56**
(13) Maintaining a conversation with someone I’ve just met	0.44	0.18	0.15	−0.06	**0.47**
(15) Greeting each person at a social meeting when I don’t know most of them	0.39	0.12	−0.03	0.16	**0.62**
(17) Talking to people I don’t know at a party or a meeting	0.53	−0.02	−0.02	0.19	**0.56**
(19) Looking into the eyes of someone I have just met while we are talking	0.37	0.37	0.19	−0.15	**0.30**
(22) Attending a social event where I know only one person	0.17	0.2	0.28	0.006	**0.58**
*Eigenvalue*	9.16	2.44	1.66	1.53	1.23
*Variance observed*	30.55	8.16	5.55	5.12	4.11

F_1_: Speaking in public/ talking with people in authority, F_2:_ Interaction with the opposite sex, F_3:_ Assertive expression of annoyance, disgust or displeasure, F_4_: Criticism and embarrassment, F_5_: Interactions with strangers

### Internal consistency

The internal consistency of the SAQ-A30 as assessed by the Cronbach’s alpha coefficient showed satisfactory results. The Cronbach’s alpha coefficient was 0.92 (Table 5).

**Table 5:** Descriptive statistics for the SAQ-A30

	Number of items	Mean (SD)	Cronbach’s alpha
1.Speaking in public/ talking with people in authority	6	16 (4.9)	0.81
2. Interaction with the opposite sex	6	16.9 (5.7)	0.84
3. Assertive expression of annoyance, disgust or displeasure	6	16.2 (4.4)	0.74
4. Criticism and embarrassment	6	18.2 (4.6)	0.74
5. Interactions with strangers	6	14.3 (4.6)	0.77
Total	30	81.8 (19.2)	0.92

## Discussion

Social anxiety is a disabling disorder that interferes significantly in many aspects of individuals’ life. The present study was designed to examine the reliability and validity of the Persian version of the SAQ-A30 in Iran. In general, this paper reported promising results. The reliability as measured by the internal consistency was found to be satisfactory. Significant correlations were observed between the SAQ-A30, the SPIN and the BFNE-S lending support to its convergent validity.

As indicated in previous research [25–27] the factor analysis of the SAQ-A30 loaded five pre-defined dimensions indicating that the factor structure of the Iranian version of the questionnaire was very similar to the original instrument. To the best of our knowledge at present the SAQ-A30 does not exist in other languages expect than an Arabic translation of the questionnaire that was used without performing any psychometric evaluation [37] and the English version of questionnaire that was used in Bangalore, India [38]. However, more recently the result for the French translation and validation study of the questionnaire was reported [39].

Evidence suggest that women are more prone to social anxiety than male gender and thus studies showed that they score significantly higher on measures that assesses social anxiety. Thus as expected the findings from this study showed that the questionnaire had reasonably a good discriminant validity. In fact female respondents scored higher than male respondents demonstrating that the questionnaire was able to differentiate well between male and female respondents. This is in line with recent finding reported by other investigators [24–27], where women showed significantly higher level of social anxiety. A recent review of the literature reported that although women experiencing higher levels of social anxiety, ‘the course of SAD seems to be similar for men and women’. The authors suggested that further integrations using existing theories are needed to better understand gender differences in SAD [7]. However, one might argue that higher social anxiety in women could be related to their biological system (hormonal fluctuation), social role, affect, perception, environment and the culture where they live [7, 39, 40].

The current study had certain limitations. The sample only included university students and non-clinical individuals that limit the generalizability of findings to the community, particularly to the clinical samples. Thus it is essential to repeat the present study and support the validity and reliability of the SAQ-A30 in a clinical setting. In addition social anxiety was only evaluated by self-reported measures. It seems that structured interviews or clinical examinations would be helpful to confirm the findings that also could help to provide cut-off values for the questionnaire. Furthermore the stability of the questionnaire was not assessed. We did assessed convergent validity by performing correlation between the SAQ-A30, the SPIN and the BFNE-S that are generic questionnaires. It was better to perform such correlation by using more specific measures of social anxiety. Finally we used the English version of the questionnaire while the original questionnaire is in Spanish. Perhaps, for certainty, it would have been better to use the original Spanish version of the questionnaire for this psychometric study.

## Conclusion

The current validation study proved that the Iranian version of SAQ-A30 has an acceptable internal consistency, and discriminative and concurrent validity and now could be used for measuring social anxiety in Iran and other Persian speaking countries. However further psychometric properties of the questionnaire including confirmatory factor analysis, and stability needs to be confirmed.

## Supplementary information

**Additional file 1.** Written informed consent.

## Data Availability

The data are available on request from the corresponding author.

## Abbreviations

SIAS: Social Interaction Anxiety Scale
SAD: Social anxiety disorder
SAQ-A30: Social Anxiety Questionnaire for Adults
SPIN: Social Phobia Inventory
BFNE-S: Brief Fear of Negative Evaluation-Straight forward items
LSAS: Liebowitz Social Anxiety Scale
SPAI: Social Phobia and Anxiety Inventory
SPS: Social Phobia Scale
BSPS: Brief Social Phobia Scale
