# Supplementary material for: Psychometric properties of the Persian version of social anxiety questionnaire for adults (SAQ-A30)
Source: Health Qual Life Outcomes. 2020 Jun 29;18:206. doi: 10.1186/s12955-020-01457-2 (PMC7325092; doi:10.1186/s12955-020-01457-2)
Supplement: Supplementary file 1 — Additional file 1. Written informed consent. [file 12955_2020_1457_MOESM1_ESM.doc]

**اجازه نامه آگاهانه از دانشجویان**

**عنوان پژوهش: اعتبار یابی نسخه فارسی پرسشنامه اضطراب اجتماعی بزرگسالان (SAQ-A3)**

**اجرا کنندگان طرح:** مهدیه موسی الرضایی – دکتر آزاده طاولی – دکتر علی منتظری

اینجانب .............................................................. دانشجوی دانشگاه ................................................................... از اهداف مطالعه فوق الذکر توسط خانم موسی الرضایی مطلع شده و با آگاهی کامل از قصد محققان در این مطالعه شرکت کرده و مشروط بر آنکه مشخصات فردی اینجانب نامعلوم و محرمانه بماند با انتشار نتایج آن موافقم.

امضاء

تاریخ

**Informed Consent Form**

**Project Title:** Psychometric properties of the Persian version of Social Anxiety Questionnaire for Adults (SAQ-A30)

**Researchers:** Mahdieh Mosarezaee, Azadeh Tavoli, Ali Montazeri

I ............................................... a student from university of ……………………………………... have been informed of the objectives of the above study by Ms. Mosarezaee and have participated in this study with full knowledge of the researchers' intentions, and I agree with the publication of its results, provided that my personal details remain unknown and confidential.

Signature

Date
